# The Impact of Fiber from Buckwheat Hulls Waste on the Pasting, Rheological and Textural Properties of Normal and Waxy Potato Starch Gels

**DOI:** 10.3390/polym13234148

**Published:** 2021-11-27

**Authors:** Greta Adamczyk, Magdalena Krystyjan, Mariusz Witczak

**Affiliations:** 1 Department of Food Technology and Human Nutrition, Institute of Food Technology and Nutrition, University of Rzeszow, Zelwerowicza Street 4, 35-601 Rzeszow, Poland; 2 Department of Carbohydrates Technology and Cereal Processing, Faculty of Food Technology, University of Agriculture in Krakow, Balicka Street 122, 30-149 Krakow, Poland; magdalena.krystyjan@urk.edu.pl; 3 Department of Engineering and Machinery for Food Industry, Faculty of Food Technology, University of Agriculture in Krakow, Balicka Street 122, 30-149 Krakow, Poland; rrwitcza@cyf-kr.edu.pl

**Keywords:** potato starch, buckwheat hulls, fiber, agriculture by-products, rheological properties

## Abstract

The aim of this study was to investigate the impact of fiber from buckwheat hull waste (BH) on the pasting, rheological, and textural properties of 4% and 5% (*w*/*w*) pastes and gels based on the potato starches with different amylose/amylopectin contents. The starch and starch/fiber mixtures were characterized by pasting and flow measurements as well as by viscoelastic and textural analysis. The pasting properties showed a greater BH effect (0.2%) on the gelatinization of PS than WPS. The starch gels and starch fiber mixtures showed biopolymer gel behavior. In the WPS/BH pastes, a smaller increase in hardness was noted compared to PS/BH.

## 1. Introduction

Starches belong to the hydrocolloid family and are used in a wide range of products either as raw materials or as food additives. Due to their amylose content, native starches have undesirable properties, such as gel retrogradation or syneresis during storage, which limits its applicability [1].

The food industry is now focused on designing new foods that are based on natural and health-promoting raw materials. In such foods, an additional advantage may be the use of by-products incurred from the agri-food processing. Changes in food texture can be achieved by adding hydrocolloids or dietary fibers, which, in small quantities bind water and can the control structure and texture of stored products [2,3,4].

From a technological point of view, dietary fiber is one of the most preferred additives in the food industry. Due to their water binding capacity, gel forming ability, texturizing and thickening effects, fiber can improve the texture, sensory characteristics, and shelf-life of foods with starchy ingredients [5]. Dietary fiber consists of two fractions: soluble and insoluble. The soluble fraction is mainly composed of resistant oligosaccharides and has a degree of polymerization (DP) that ranges from 3 to 9 and viscous fibers that have a high molecular weight [6]. The insoluble fraction includes cellulose, hemicelluloses, and lignin. The content of the soluble and insoluble dietary fiber in different foodstuffs varies: apple fiber (13.9 g/100 g SDF and 48.7 g/100 g IDF), oat fiber (1.5 g/100 g SDF and 73.6 g/100 g IDF), and tomato fiber (8.3 g/100 g SDF and 57.6 g/100 g IDF) [7]. According to Matseychik et al. (2020) [8] and Korpacheva et al. (2021) [9], there is a significant amount of fiber in buckwheat hull, with contents reaching up to 80%.

The hulls of the buckwheat seeds that are produced during the de-hulling process are a waste material and are burned after harvest. In areas where buckwheat is cultivated, this is an environmental problem [10]. Recently, there has been increased interest in renewable by-products as promising raw materials that can be used in the production of a variety of food additives, chemicals, textiles, building materials, biotechnologies, and medicines because they are cheap and abundant agricultural bioproducts. The analytical characteristics of the buckwheat hemicelluloses show that they are a rich source of glucuronoxylan-type hemicelluloses (mainly composed of 4-o-methylglucuronoxylan) [8,9,11,12,13,14].

The addition of natural fibers to starches influences their properties. Different fibers have been added to starches, such as those from wheat, cotton, oat, apple, pea, coir, vegetal, or barley-glucan [2,15,16].

Due to its composition and characteristics, buckwheat waste has the potential to be an interesting raw material. To the best of our knowledge, the effects of buckwheat fiber sourced from hulls on potato starch gels have not been studied. This study aims to determine the potential of agricultural by-products such as buckwheat fiber to be used in starch-based foodstuffs. The hypothesis is that buckwheat hulls, as an agricultural by-product, can be used as an additive in starch-based foods to enrich products that already have a high fiber content. For this purpose, mixtures of two types of starch (normal potato starch and waxy potato starch) with dietary fiber from roasted buckwheat hulls (BH) will be prepared. Starch pastes and gels at two concentrations of 4% and 5% (*w*/*w*) will be used as reference samples. The amount of fiber that will be added will be fixed.

## 2. Materials and Methods

### 2.1. Materials

The starches that were used in the study were normal potato starch (PS) of superior standard (WPPZ S.A., Luboń, Poland) and waxy potato starch (WPS) Eliane 100 (AVEBE FOOD, Veendam, The Netherlands). According to AOAC (2006) [17], the PS had the following parameters: 80.97% dry mass (d. m.); 29% amylose; 111 mg/100 g total phosphorus; 0.20 g/100 g protein; 0.10 g/100 g lipids. The WPS contained 84.50% d. m., <1% amylose, 82 mg/100 g total phosphorus, 0.25 g/100 g protein, and 0.11% lipids.

Ecological dietary fiber separated from roasted buckwheat hulls (BH) was assessed according to the methods outlined in AOAC (2006) [17] and were determined to have the following parameters: 89.02% d. m.; lipids 0.60 g/100 g, carbohydrates 3.10 g/100 g, and protein 4.50 g/100 g. The hulls were purchased and manufactured in Poland (Look Food, Lublin, Poland). The total dietary fiber (TDF) in the buckwheat hull was evaluated using the enzymatic method [17] and amounted to 71.40 g/100 g and included the amount of insoluble dietary fiber (IDF) 61.06% and soluble dietary fiber (SDF) 10.34%.

### 2.2. Methods

#### 2.2.1. Sample Preparations

Dietary fiber was separated from the roasted buckwheat hulls (BH) and starch: normal potato starch (PS) and waxy potato starch (WPS) were used. The control samples were starch pastes and gels at two concentrations (4% and 5% *w*/*w*). Starch + BH mixtures were prepared in the following proportions: 3.8 + 0.2 and 4.8% + 0.2% (*w*/*w*).

Aqueous solutions of starch and starch–fiber mixtures were heated using the Viscograph-E Brabender. Samples that were prepared in the Viscograph were also used for textural analysis.

For rheological analysis (flow curves and oscillatory measurements), the aqueous suspensions of the starches (4% and 5% *w*/*w*) and the starches (3.8% and 4.8% *w*/*w*) with the fiber from the buckwheat hulls (0.2% w/w) were agitated (with constant stirring 400 rpm) for 10 min at room temperature and were further agitated at 95 °C for 30 min. The samples that were obtained were distributed to the device’s measuring system.

#### 2.2.2. Pasting Characteristics

The measurements of the pasting characteristics of normal and waxy potato starches (4–5% *w*/*w* d. m.) and mixtures of starches (3.8% and 4.8% *w*/*w* d. m.) with buckwheat hulls (BH) (0.2% *w*/*w* d. m.) were taken using the Viscograph-E Brabender (Brabender GmbH & Co. KG, Duisburg, Germany). The aqueous suspensions of the potato starch blends were prepared at ambient temperature, and pasting behavior was measured using the viscograph for 83 min.

The measurements of the pasting characteristics of the PS and WPS suspensions as well as the mixtures of PS/BH and WPS/BH were run with following parameters:

(a) Heating from 30 to 95 °C heated at 1.5 °C/min;
(b) Maintaining samples for 5 min at 95 °C;
(c) Cooling 95 to 50 °C with 1.5 °C/min;
(d) Constant agitation at 75 rpm.

Measurements were run in duplicate. Based on the analysis, the following parameters were obtained: *T*_0_ (°C)—temperature at the beginning of pasting; *η_max_* (Brabender units (BU))—maximum viscosity; *T_ηmax_* (°C)—temperature at maximum viscosity; *η_95°C_* (BU)—viscosity at 95 °C; *η_95°Cafter5min_* (BU)—viscosity at 95 °C after 5 min holding; *η_min_*_._ (BU)—minimum viscosity; *breakdown (BD)* (BU); and *η_50°C_* (BU)—viscosity after cooling to 50 °C. Viscograph Data Correlation software was used to process the data (Brabender GmbH & Co. KG, Duisburg, Germany).

#### 2.2.3. Flow Curves

Flow curves were created using a RS6000 (Gebrueder Haake GmbH, Karlsruhe, Germany) rheometer in controlled rate of shear (CR) mode with a CC26 Ti measuring system and a 1.9 gap. Measurements were taken at a constant temperature of 50 °C. The shear rate was raised from 0 to 300 s^−1^ for 10 min and was maintained at the maximum shear rate for 1 min; it was then decreased from 300 to 0 s^−1^ over the course of 10 min.

The up (the shear rate from 0 to 300 s^−1^ within 600 s) and down (the shear rate from 300 to 0 s^−1^ within 600 s) flow curves were fitted to an Ostwald–de Waele (1) model [18].

(1) $$\tau =K\cdot {\dot{\gamma }}^{n}$$

where *τ*—shear stress (Pa), *K*—consistency coefficient (Pa∙s^n^), $\dot{\gamma }$—shear rate (s^−1^), and *n*—flow behavior index (-).

The areas of the thixotropy and anti-thixotropy hysteresis loops were calculated according to Sikora et al. (2015) [19].

#### 2.2.4. Oscillatory Measurements

Mechanical spectra measurements were taken with the RS6000 rheometer (Gebrueder Haake GmbH, Karlsruhe, Germany) in oscillatory mode and had a parallel plate geometry (60 mm diameter) and a 1.0 mm gap. The small oscillatory shear test was performed at 25 °C in a sweep frequency range of 0.1–10 Hz, which was within the range of the linear viscoelasticity region for all of the samples. The mechanical spectra—storage modulus (*G′*), loss modulus (*G″*), and loss tangent (tan *δ*)—were measured. The mechanical spectra were run in duplicate.

#### 2.2.5. Textural Properties

Texture was characterized with a Texture Analyzer EZ-Test EZ-LX (Shimadzu, Japan). The textural properties of the starches and their blends with the addition of buckwheat hulls were determined using the penetration test. The freshly obtained gels in Viscograph-E (about 30 mL) were transferred to plastic cylinders. Texture measurements were performed for fresh gels (after 1 h of storage) and after 1 day and 7 days of storage at 4 °C. The samples were compressed with a cylinder probe (ø 7.0 mm) at 50 mm/min. speed. Gel hardness was defined as the maximal force applied (N). The reported results were the average values of at least three replications [2].

#### 2.2.6. Statistics

The experimental data were calculated using Statistica v.13.3 (StatSoft, Inc., Tulsa, OK, USA). For all determinations, one-way ANOVA was performed using Duncan’s test at the confidence level of α = 0.05.

## 3. Results

### 3.1. Pasting Properties of Starch/Fiber Mixtures

In Table 1 and Table 2, the mean values for the pasting properties are summarized for the native (PS) and waxy (WPS) potato starch as well as for their blends with the buckwheat hulls (BH). It is well known that the parameters of the pasting characteristics strongly depend on the starch concentration [19,20]. With the increasing starch concentration (from 4% to 5% (*w*/*w*)), the decrease in the gelatinization temperature (*T*_0_) and temperature at the maximum viscosity (*T_ηmax_*) were observed. However, the starch concentration did not affect the temperature at the minimum viscosity (*T_ηmin_*), which was also confirmed by Adamczyk et al. (2020) [2] and Krystyjan et al. (2015) [21].

PS and WPS (3.8–4.8%) samples with 0.2% *w*/*w* BH developed higher *T_0_* and *T_ηmax_* values than the samples that only contained potato starch. An increase in *T*_0_ due to the addition of compounds such as hydrocolloids or fibers was also reported by Adamczyk et al. (2020) [2], Leite et al. (2012) [22], and Yildiz et al. (2013) [15]. The presence of 0.2% BH in starch pastes was associated with a lower maximum viscosity value (*η_max_*) and lower or no statistically significant differences in viscosity at 95 °C (*η_95°C_*) compared to plain potato starch (Table 1 and Table 2). The replacement of starch with buckwheat hulls reduced the values of *η_max_* by 18.9% (4% PS) and 28.2% (5% PS) and by 4.2% (4% WPS) and 6.2% (5% WPS). Such a tendency indicates a reduction in the swelling rate of the native starch granules, something that was also claimed by Ragaee and Abdel-Al. (2006) [23]. In the present study, BH markedly decreased the viscosity of native starch but affected the maximum viscosity of waxy starch to a lesser extent. The decrease in the *η_max_* value of waxy potato starch in the presence of fiber was observed in our previous study [2]. The addition of apple and oat fiber resulted in decreased viscosity, but the impact was definitely greater than it was for BH. The differences were affected by the variable content of soluble and insoluble dietary fiber. Yildiz et al. (2013) [15] explained that starch with a higher swelling capacity resulted in a higher maximum viscosity during pasting/heating. The increased of the swelling capacity in the starch/fiber mixture may come from the water-insoluble fiber fraction, which the caused disruption in the amylopectin structure. Insoluble dietary fiber (IDF) has a tendency for competitive hydration due to the disruption of the starch matrix during gelatinization. The dietary fiber used in this study contained an insoluble fraction of 10.34%, and it was more than it was in oat and tomato fiber [7].

The minimum viscosity values (*η_min_*) of blends with BH that also had a lower starch content (4%) was increased with BH was added, but conversely, at higher concentrations (5%)—*η_min_* decreased.

It was determined that the viscosity of the WPS and WPS/BH pastes did not increase greatly in the tested samples after then had been cooled to 50 °C (*η_50°C_*) in relation to the *η_min_*. The *η_min_* values for WPS/BH were close to the viscosity for the control sample (WPS). In the case of PS, only slight influence on the *η_50°C_* was reported after the addition of the BH. After being cooled, PS created less viscous systems in 4% PS/BH and more viscous systems in 5% PS/BH blends.

The breakdown parameter (BD) describes the tendency of starch to resist shear force during heating and the stability of the final product [17]. We noted that the presence of BH in PS mixtures decreased the BD parameter. After gelatinization, the granules of 4% and 5% PS were easily broken down, resulting in higher breakdown values (278 and 596 BU) than those determined in the samples with BH (118 and 246 BU). In the case of WPS, the presence of BH in the 4% blends did not significantly change this parameter (594 and 546 BU). This parameter was also not significantly changed in the 5% systems, but there were slight differences (903 and 838 B.U.). Our earlier research indicated that the presence of 0.2% apple and oat fiber in mixtures with 3.8% waxy potato starch significantly decreased BD [2]. However, Yildiz et al. (2013) [15] claimed that fiber preparations from apples, oats, and lemons increased the *BD* value and that when the impact was more pronounced, then it usually reflected the fact that a higher concentration of the component was used. The BD results show that the addition of BH had an influence on the stability of the PS but not on the stability of the WPS. According to Burisova et al. (2002) [24], the glucuronoxylan from buckwheat hulls prevents the retrogradation of starch and represents a potential additive in various corn starch-based food products.

Buckwheat fiber had a greater effect on the gelatinization profile of native potato than it did on waxy starch. Similar results were observed in our previous work [2], where apple fiber was added to waxy potato starch. It was confirmed that some hydrocolloids and fibers alter the pasting and hydration behavior of starch granules, and the extent of the modification was dependent on the type and level of the hydrocolloid, fiber, and the starch origin [21,25,26]. These additives caused interference during the pasting process of the starch granules. As a result, the reduction in the breakdown and setback parameter of the native starch paste and the re-ordering of the amylose matrix after leaching from the granules were observed [27,28,29]. It can therefore be assumed that the presence of dietary fiber in the mixture with starch inhibited the starch gelatinization process, resulting in less amylose being leached from the starch granules. This behavior could be due to the competition between starch and dietary fiber for water molecules during the heating process of the mixtures. Our assumption was confirmed by the obtained results. In each of the cases that were studied, the initial gelatinization temperature increased and the maximum viscosity decreased after the addition of the BH to the mixture of native potato starch, causing the starch granule to swell less and the gelatinization of the starch to be reduced. The results are different from those reported by Yildiz et al. (2013) [15]. The authors, while studying the impact of oat, pea, lemon, and apple fiber additives on the pasting characteristics of wheat starch, observed a decrease in the *T_0_* and an increase in the *η_max_* of starch blends against a control sample (fiber-free). Such a distinct influence of fiber on the rheological properties of starch results, among other factors, from differences in the content of soluble (SDF) and insoluble fractions (IDF) in the fiber. In the case of fiber applied by Yildiz et al. (2013) [15], TDF constituted 90%, where the insoluble fraction accounted for 50% of the apple fiber and up to 85% for the oat fiber. The insoluble fiber (IDF)consisting mainly of cellulose, lignins, and certain non-cellulosic polysaccharides, causes the disruption of the starch granules during heating, which in turn releases more amylopectin into the system [15,30,31,32].

### 3.2. Rheological Properties of Starch/Fiber Mixtures

#### 3.2.1. Flow Curves Properties

**Figure 1:**
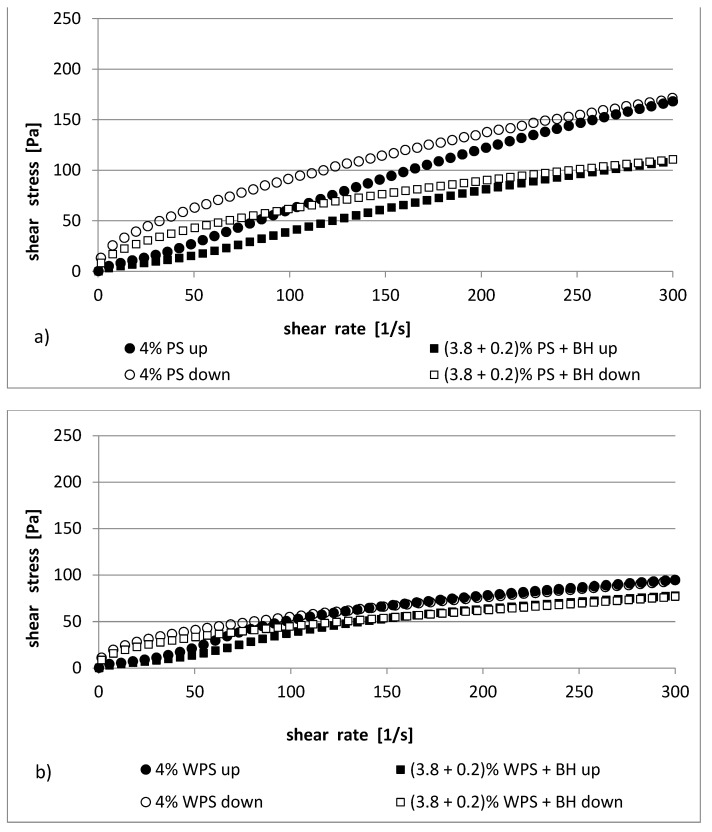
Flow curves of (**a**) 4% PS pastes and their mixtures with BH; (**b**) 4% WPS pastes and their mixtures with BH.

**Figure 2:**
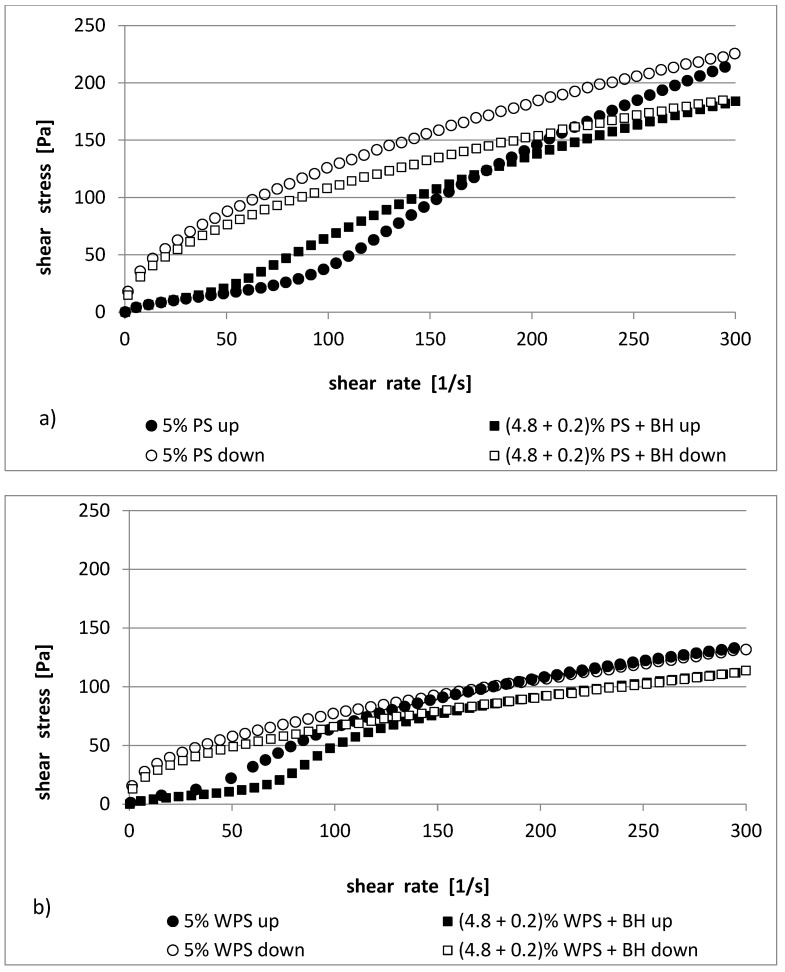
Flow curves of (**a**) 5% PS pastes and their mixtures with BH; (**b**) 5% WPS pastes and their mixtures with BH.

The parameters of the rheological model that was used to describe the experimental curves are provided in Table 3 and Table 4. In Figure 1 and Figure 2, the flow curves of potato starch (native and waxy) in the presence or absence of BH are shown.

The studied pastes exhibited non-Newtonian, shear-thinning properties. The flow curves that were determined at increasing (shear rate from 0 to 300 s^−1^ within 600 s) and decreasing (shear rate from 300 to 0 s^−1^ within 600 s) shear rates were able to be fitted to an Ostwald–de Waele model very well (R^2^ = 0.977–0.999).

The consistency coefficient (*K*) of the PS and WPS increased with the paste concentration (4% and 5%). However, the presence of buckwheat hulls in the starch pastes decreased this value by about 50%. For example, the 4% blends PS and PS/BH had 0.96 and 0.51 Pa∙s^n^, whereas the 4% blends of WPS and WPS/BH had 2.06 and 1.07 Pa∙s^n^, respectively (in the range of increasing shear rates from 0 to 300 s^−1^) (Table 3). A similar relationship was observed in the case of the 5% mixtures (Table 4). In the range of the decreasing shear rates (from 300 to 0 s^−1^), there was tendency for the impact of the buckwheat hulls to be similar, with the main difference being that the coefficient *K* was characterized by higher values (e.g., 4% PS and PS/BH—7.38 and 5.49 Pa∙s^n^, respectively).

The flow index (*n*) for the up curves showed shear-thinning properties (*n* < 1) for both the increasing and decreasing shear rates. The shear-thinning behavior was common for starch pastes [25,33,34]. The flow index increased in the 4% PS/BH (0.95) and WPS/BH (0.77) pastes compared to the control samples (PS—0.92, WPS—0.69). However, in the case of a higher starch concentration, an inverse relationship was demonstrated in one of the 5% PS samples; there was a decrease in the value of the *n* parameter, and there was a non-significant increase in the 5% WPS.

The starch pastes showed hysteresis loop (expressed as the area between the “up” and “down” flow curves) (Figure 1 and Figure 2). The curves showed either a clockwise or anticlockwise course (thixotropic or anti-thixotropic behavior). The normal potato starch and their blends with BH were characterized with one type of loop (anticlockwise) (Figure 1a and Figure 2a), while in the waxy potato starch samples and in the samples of WPS with BH, they had a mixed characteristic (Figure 1b and Figure 2b)—clockwise/anticlockwise course. The intersection of the flow curves of the WPS and WPS/BH pastes was observed at higher shear rates (shear rate higher than 100 s^−1^). The values of the thixotropy and anti-thixotropy hysteresis loop areas (the work where the internal structure of the studied sample is destroyed and rebuilt) were calculated by summing the areas of particular trapeziums between the “up” and “down” hysteresis loop curves and are presented in Table 3 and Table 4. Native 4% starch paste was characterized by total surface area values (466.8 Pa/s) that were twice as high as those for waxy starch (195.9 Pa/s). A similar relationship was observed in their mixtures with BH (471.3 and 207.1 Pa/s, respectively). However, the addition of BH into the 4% native and waxy starch pastes as a replacement for 0.2%, was not statistically significant. In the case of the 5% PS pastes, the area of the hysteresis loop was three times larger (1180.9 Pa/s) than it for the 5% WPS (382.9 Pa/s) pastes. The presence of BH in the mixtures caused a decrease in the area value of the loop in the native starch by 50% (510.4 Pa/s), whereas in the case of waxy starch, it did not matter (329 Pa/s).

In both the native and waxy potato starch pastes, the replacement of 0.2% starch with the addition of BH caused a reduction in the shear stress (Figure 1 and Figure 2). The shear stress values showed a greater tendency to decrease in the PS/BH pastes (Figure 1a and Figure 2a) compared to the WPS/BH pastes (Figure 1b and Figure 2b). This means that the addition of buckwheat hulls to starch pastes caused greater rheological stability in the PS/BH systems than it did in the WPS/BH systems compared to the control samples (PS, WPS). The thixotropic and anti-thixotropic behavior of starch dispersions and their mixtures with additives such as hydrocolloids or dietary fibers has already been observed for various kinds of starch [2,35,36]. This phenomenon depends on the type and concentration of starch, the additives that are present in the starch blends (hydrocolloids, fibers, etc.), or the range of the shear rates [20,36,37].

#### 3.2.2. Viscoelastic Properties

**Figure 3:**
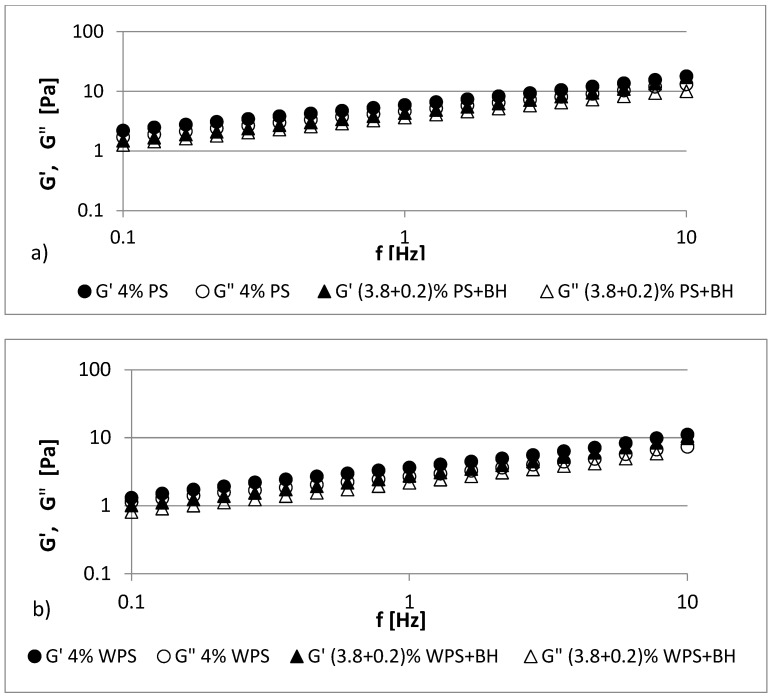
Mechanical spectra (storage modulus (*G′*) and loss modulus (*G”*)) of (**a**) 4% PS gel and its mixture with BH; (**b**) 4% WPS gel and its mixture with BH.

**Figure 4:**
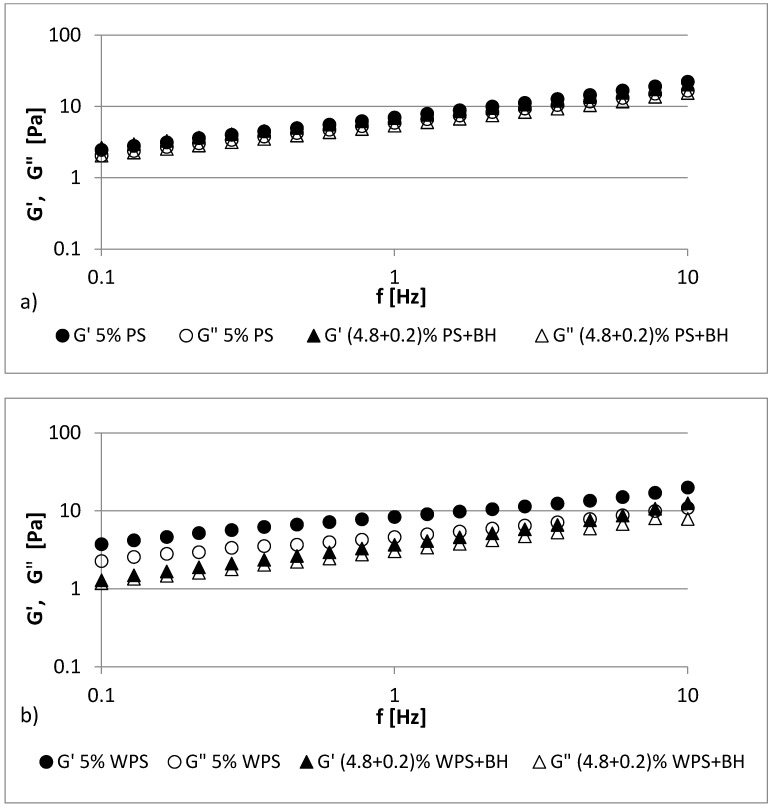
Mechanical spectra (storage modulus (*G′*) and loss modulus (*G”*)) of (**a**) 5% PS gel and its mixture with BH; (**b**) 5% WPS gel and its mixture with BH.

The frequency sweep oscillatory curves of the fresh native and waxy potato starch gels with BH at 25 °C are shown in Figure 3 and Figure 4. The studied 4–5% starch gels (PS, WPS) and all of the starch–hulls mixtures showed typical biopolymer gel behavior, wherein the *G′* value exceeded the *G”* value in the whole frequency sweep range. In the starch gels, both of the moduli were frequency dependent because the values of *G’* and *G”* over the whole frequency range increased when the angular frequency increased [38]. However, the storage and loss values of the moduli were very close to each other, and *G’* showed a spectrum that was parallel to *G”*; moreover, no crossover was observed between them throughout the experiment when the frequency varied from 0.1 to 10 Hz (Figure 3 and Figure 4). The granular structure of the starch and the amylose content were responsible for the differences in the rheological behaviors [39].

Additives such as hydrocolloids or fiber modify the dynamic spectra of starch, and different trends can be observed [40]. The presence of BH in starch pastes caused a decrease in *G’* and *G”* values to a small extent, especially when compared to those of the 4% PS and WPS pastes (Figure 3a,b). In the case of the 5% samples, a similar relationship was observed, but more significant changes were observed in the values for the 5% WPS paste (Figure 4a,b). These results indicate that potato starch gels both with and without BH can be classified as having a typical weak gel structure. In all cases, elastic properties prevailed over viscous ones (*G’* > *G”*). In general, the replacement of starch with the addition of buckwheat hulls slightly reduced the values of *G′* and *G″* compared to the control samples (PS, WPS).

**Figure 5:**
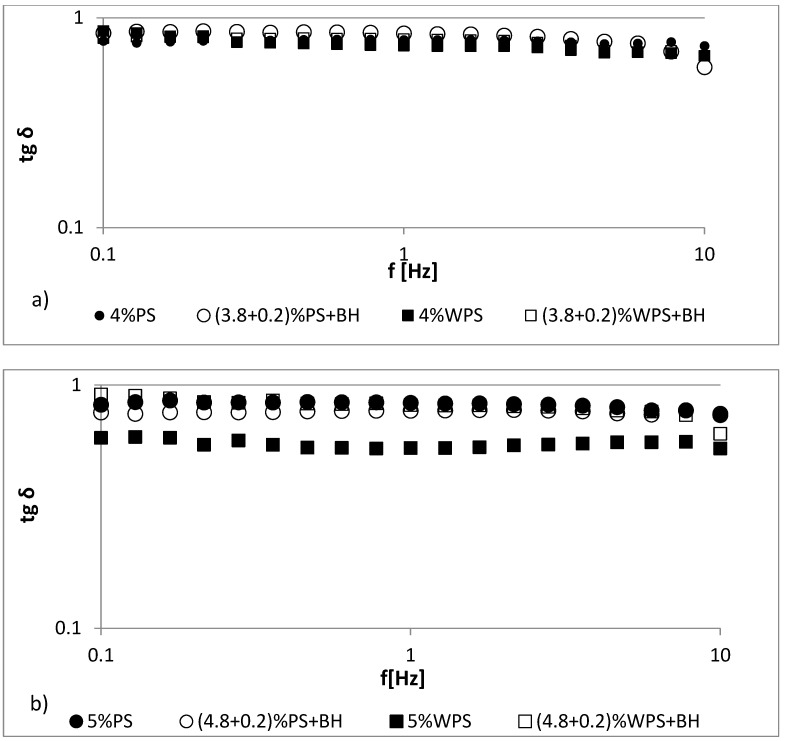
The loss tangent (tan δ) of (**a**) 4% PS and 4% PS/BH pastes; (**b**) 5% PS and 5% PS/BH pastes.

The loss tangent (tan *δ*) is a rheological parameter that is associated with the energy lost per cycle divided by the energy stored per cycle (*G″/G′*). In this way, values of tan *δ* < 1 indicate that the behavior of the material is elastic and tan *δ* > 1 indicates viscous properties [38,41]. The tan *δ* values for all studied gels were smaller than 1, indicating predominantly elastic behavior (Figure 5). The dynamic mechanical loss tangent (tan *δ*) of the 4% normal and waxy potato starch-BH gels was higher than tan *δ* of the plain starch gels. The same tendency was observed for 4% waxy potato starch-BH mixture, but the effect was more predominant. These results indicated that 4% PS/BH, 4% WPS/BH and 5% WPS/BH mixtures were less elastic than control samples (plain starch gels). The values of tan *δ* being close to 1 at this frequency suggested that samples were more liquid-like, because the values of *G′* and *G"* was very close from each other. An opposite effect was observed for the 5% native potato starch–BH sample; the tan *δ* of the starch-BH gels was lower than the tan *δ* of the control sample (5% PS). The effect was more pronounced in the frequency range between 0.1 and 1.0 Hz. A less pronounced effect was noted at higher frequencies (between 1.0 and 10 Hz), where the tan *δ* for the starch pastes and their mixtures with BH was at the same level. These results indicate that the 5% native starch–BH gel was more elastic than the control sample.

### 3.3. Textural Properties of Starch/Fiber Mixtures

The textural changes of the starch-based gels with and without BH during storage are presented in Table 5 and Table 6 and indicate that the addition of buckwheat hulls had an effect on the hardness values of the native and waxy potato starch.

It was found that the hardness of the tested gels was affected by the type and concentration of the starch that was used. As the starch concentration increased, the hardness values of the starch-alone and starch/buckwheat fiber gels increased. In all of the studied cases (except 5% WPS), the hardness of the gels was higher than that of the fresh gels after 1 day of storage, independent of the starch concentration.

The effect of BH fiber on the hardness values of the gels was not statistically significant for the fresh gels (H_0_). The hardness values (H_1_, H_7_) of the gels increased with the storage time (1 day and 7 days), but the effect was more significant in the case of the native starch. The PS-alone gels (4% and 5%) showed a more noticeable increase in the gel hardness over time than the WPS-alone gels.

We observed that replacing the starch with BH affected the gel hardness values after 1 day and 7 days of storage in the case of native starch compared to waxy potato starch. This tendency was observed for both the 4% and 5% starch concentrations. The gel hardness increase was associated with the amylose and amylopectin content and occurred due to a process called retrogradation [42]. According to Funami et al. (2005) [43] and Krystyjan et al. (2013) [4], amylose is responsible for short-term changes (up to 48 h) in the structure of the starch gel. In turn, amylopectin is responsible for long-term starch retrogradation and can even last several weeks. On the other hand, the rate of the retrogradation process in such starches is higher than it is in starches that contain little or no amylose [42,44]. The results of the textural parameters confirm these assumptions (Table 5 and Table 6), similar to the data that were obtained for the pasting characteristics. Comparing the viscosity of the starch pastes after cooling, it can be clearly seen that in the case of native potato starch, the increase of this parameter was much larger than it was for waxy starch pastes with a low amylose content. As noted by Dobosz et al. (2019) [45], the greater the increase of this parameter, the greater the tendency of starch to undergo retrogradation.

The addition of fiber reduced the rate of starch retrogradation because a much smaller increase in hardness was noted compared to native plain starch gel after 1 day of storage (36.6% lower for the mixtures with a 4% concentration and 20.4% lower for the 5% mixtures) (Table 5 and Table 6). In the case of the waxy starch, the effect of the BH additive was much smaller. On this basis, it can be concluded that SDF because is a substance that has a strong affinity for water, it competed with the starch; in particular, it competed with amylose, hindering its pasting process. Consequently, a smaller amount of amylose flowed from the starch granules, and during the cooling phase, the paste thus underwent less retrogradation.

## 4. Conclusions

The rheological and textural properties of potato starch pastes and gels were influenced by buckwheat hull fiber. The presence of BH in mixtures comprising native potato starch increased the initial pasting temperature and decreased the maximum viscosity, leading to the starch granule swelling less and the gelatinization of the starch becoming reduced; as a result of this, less amylose leached from the starch granules. The antagonistic interaction between BH and amylose was confirmed by examining the impact of fiber on the rheological properties of WPS. In cases where there was a limited amount of amylose, the effect of the added fiber turned out to be much smaller.

The studied pastes exhibited non-Newtonian, shear-thinning properties. The flow curves at the increasing (shear rate from 0 to 300 s^−1^ within 600 s) and decreasing (shear rate from 300 to 0 s ^−1^ within 600 s) shear rates were able to be fit to an Ostwald–de Waele model very well and showed loop hysteresis. The normal potato starch and the blends of normal potato starch with BH were characterized with one type of loop (anticlockwise), while in the waxy potato starch samples and WPS with BH had a mixed characteristic—clockwise/anticlockwise course. On the other hand, the addition of buckwheat hulls to the starch pastes showed smaller areas between the flow curves; therefore, these PS/BH pastes showed greater rheological stability than the WPS/BH systems did, especially when compared to the control samples (PS, WPS). Native starch paste (4%) was characterized as having total surface area values (466.8 Pa/s) that were twice as high as those of waxy starch (195.9 Pa/s). In the case of the 5% PS pastes, the area of the hysteresis loop was three times larger (1180.9 Pa/s) than it was for the 5% WPS pastes (382.9 Pa/s). The rheological results that were obtained showed that the native starch–BH gel was more elastic than the control sample. In the WPS/BH gels, a smaller increase was noted in the hardness value compared to PS/BH gels, suggesting the influence of BH on the starch retrogradation process. In summary, BH had a greater effect on the pasting, flow, and textural properties of native potato starch than it did on waxy starch and showed stronger interaction with amylose than with amylopectin. Our results indicate that buckwheat hulls, an agricultural by-product, can be used as an additive in starch-based food to enrich the amount of fiber that is present in the product. Therefore, this research may be important for the design of starch-based foods that are enriched with TDF by-products and that have a desirable texture and high storage stability as well as foods with beneficial health properties. Moreover, interesting rheological properties create the possibility for the mixtures obtained in this study to be used in a wide range of applications, e.g., in the development of biodegradable composites, which may be used as films, coatings, and other packaging materials in the future.

## Figures and Tables

**Table 1:** Pasting characteristics of 4% PS and WPS with addition of 0.2% BH.

Sample	T_0_ (°C)	*η_max_* (BU)	T*_ηmax_* (°C)	*η_95°C_* (BU)	*η_95°Cafter5min_* (BU)	*η_min_* (BU)	BD (BU)	*η_50°C_* (BU)
4% PS	63.5 ± 0.07 ^a^	878.0 ± 5.66 ^b^	87.7 ± 0.14 ^c^	760.5 ± 0.71 ^c^	600.0 ± 0.00 ^b^	512.0 ± 1.41 ^b^	278.0 ± 5.66 ^b^	619.5 ± 4.95 ^b^
(3.8 + 0.2)% PS + BH	65.3 ± 0.00 ^ab^	712.0 ± 8.49 ^a^	92.5 ± 0.71 ^d^	704.5 ± 6.36 ^b^	549.0 ± 2.83 ^b^	529.5 ± 7.78 ^b^	118.0 ± 11.31 ^a^	657.5 ± 0.71 ^c^
4% WPS	70.7 ± 3.89 ^b^	943.5 ± 55.86 ^b^	73.4 ± 0.07 ^a^	412.0 ± 15.56 ^a^	349.0 ± 14.14 ^a^	309.5 ± 10.6 ^a^	594.5 ± 41.72 ^c^	345.0 ± 12.73 ^a^
(3.8 + 0.2)% WPS + BH	68.5 ± 0.14 ^ab^	903.5 ± 17.68 ^b^	74.5 ± 0.14 ^b^	433.5 ± 10.61 ^a^	357.0 ± 9.90 ^a^	311.5 ± 7.78 ^a^	546.5 ± 7.78 ^c^	341.5 ± 9.19 ^a^

Parameters in columns denoted with the same letters do not differ significantly at the level of confidence α = 0.05.

**Table 2:** Pasting characteristics of 5% PS and WPS with addition of 0.2% BH.

Sample	T_0_ (°C)	*η_max_* (BU)	T*_ηmax_* (°C)	*η_95°C_* (BU)	*η_95°Cafter min_* (BU)	*η_min_* (BU)	BD (BU)	*η_50°C_* (BU)
5% PS	62.9 ± 0.00 ^a^	1364.5 ± 36.06 ^c^	80.4 ± 0.78 ^b^	962.5 ± 12.02 ^b^	768.0 ± 7.07 ^b^	665.5 ± 4.95 ^b^	596.5 ± 28.99 ^b^	826.0 ± 7.07 ^b^
(4.8 + 0.2)% PS + BH	65.1 ± 0.07 ^b^	979.0 ± 69.3 ^a^	88.6 ± 0.14 ^c^	905.5 ± 68.59 ^b^	732.5 ± 60.1 ^b^	637.0 ± 36.8 ^b^	246.5 ± 9.19 ^a^	797.5 ± 50.2 ^b^
5% WPS	67.6 ± 0.14 ^c^	1386.5 ± 0.71 ^c^	72.9 ± 0.14 ^a^	575.5 ± 3.54 ^a^	483.0 ± 5.66 ^a^	436.00 ± 1.41 ^a^	903.5 ± 6.36 ^d^	494.5 ± 0.71 ^a^
(4.8 + 0.2)% WPS + BH	67.9 ± 0.00 ^d^	1300.0 ± 11.3 ^b^	72.95 ± 0.07 ^a^	552.0 ± 4.24 ^a^	462.00 ± 5.66 ^a^	412.5 ± 4.95 ^a^	838.0 ± 5.66 ^c^	461.5 ± 9.19 ^a^

Parameters in columns denoted with the same letters do not differ significantly at the level of confidence α = 0.05.

**Table 3:** Parameters of steady flow measurements of 4% PS and WPS pastes and their mixtures with BH as well as the area values of the hysteresis loops.

Sample	Ostwald–de Waele Model						Areas of the Hysteresis Loops (Pa/s)		
	K (Pa∙s^n^)		n (-)		R^2^		A	T	A+T
	0–300 s^−1^	300–0 s^−1^	0–300 s^−1^	300–0 s^−1^	0–300 s^−1^	300–0 s^−1^			
4% PS	0.96 ± 0.21 ^b^	7.38 ± 0.43 ^c^	0.92 ± 0.04 ^c^	0.55 ± 0.01 ^c^	0.998	0.999	466.8 ^b^	-	466.8 ^b^
(3.8 + 0.2)% PS + BH	0.51 ± 0.08 ^a^	5.49 ± 0.12 ^a^	0.95 ± 0.03 ^c^	0.53 ± 0.01 ^b^	0.997	0.999	471.3 ^b^	-	471.3 ^b^
4% WPS	2.06 ± 0.15 ^c^	6.89 ± 0.28 ^b^	0.69 ± 0.01 ^a^	0.46 ± 0.01 ^a^	0.987	0.999	172.3 ^a^	23.6 ^a^	195.9 ^a^
(3.8 + 0.2)% WPS + BH	1.07 ± 0.08 ^b^	5.53 ± 0.11 ^a^	0.77 ± 0.01 ^b^	0.47 ± 0.01 ^a^	0.980	0.999	187.2 ^a^	19.9 ^a^	207.1 ^a^

Different letters within a column represent significant differences (α = 0.05). Antithixotropy—A; thixotropy—T; total area: A+T.

**Table 4:** Parameters of steady flow measurements of 5% PS and WPS pastes and their mixtures with BH.

Sample	Ostwald–de Waele Model						Areas of the Hysteresis Loops (Pa/s)		
	K (Pa∙s^n^)		n (-)		R^2^		A	T	A+T
	0–300 s^−1^	300–0 s^−1^	0–300 s^−1^	300–0 s^−1^	0–300 s^−1^	300–0 s^−1^			
5% PS	1.24 ± 0.07 ^a^	11.42 ± 0.23 ^b^	0.93 ± 0.11 ^a^	0.53 ± 0.01 ^b^	0.994	0.999	1180.9 ^c^	-	1180.9 ^c^
(4.8 + 0.2)% PS + BH	1.19 ± 0.14 ^b^	10.76 ± 0.33 ^b^	0.93 ± 0.08 ^a^	0.50 ± 0.00 ^c^	0.989	0.999	510.4 ^b^	-	510.4 ^b^
5% WPS	2.01 ± 0.24 ^c^	7.58 ± 0.43 ^a^	0.81 ± 0.09 ^b^	0.44 ± 0.01 ^a^	0.985	0.999	382.9 ^a^	7.7 ^a^	390.6 ^a^
(4.8 + 0.2)% WPS + BH	1.03 ± 0.19 ^b^	7.26 ± 0.04 ^a^	0.86 ± 0.04 ^b^	0.47 ± 0.01 ^b^	0.977	0.999	280.6 ^a^	48.4 ^b^	329.0 ^a^

Different letters within a column represent significant differences (α = 0.05). Antithixotropy—A; thixotropy—T; total area: A+T.

**Table 5:** Gel hardness (N) of 4% normal and waxy potato starch gels and their mixtures with buckwheat hulls measured on the day of preparation and after 1 day of storage.

Samples	Hardness (N)		
	H_0_	H_1_	H_7_
4% PS	0.40 ± 0.03 ^b^	2.26 ± 0.21 ^c^	2.88 ± 0.42 ^c^
(3.8 + 0.2)% PS + BH	0.43 ± 0.02 ^b^	1.54 ± 0.02 ^b^	2.08 ± 0.05 ^c^
4% WPS	0.13 ± 0.01 ^a^	0.18 ± 0.05 ^a^	0.61 ± 0.12 ^b^
(3.8 + 0.2)% WPS + BH	0.17 ± 0.01 ^a^	0.18 ± 0.01 ^a^	0.27 ± 0.04 ^a^

Parameters in columns denoted with the same letters do not differ significantly at the confidence level α = 0.05. H_0_—hardness of fresh gels (N); H_1_—hardness of gels after 24 h of storage (N); H_7_—hardness of gels after 7 days of storage (N).

**Table 6:** Gel hardness (N) of 5% normal and potato starch gels and their mixtures with buckwheat hulls measured on the day of preparation and after 1 day of storage.

Samples	Hardness (N)		
	H_0_	H_1_	H_7_
5% PS	0.60 ± 0.00 ^b^	3.85 ± 0.44 ^c^	3.63 ± 0.25 ^c^
(4.8 + 0.2)% PS + BH	0.42 ± 0.00 ^b^	2.15 ± 0.05 ^b^	2.14 ± 0.05 ^b^
5% WPS	0.18 ± 0.01 ^a^	0.18 ± 0.01 ^a^	0.94 ± 0.02 ^a^
(4.8 + 0.2)%WPS + BH	0.19 ± 0.03 ^a^	0.24 ± 0,04 ^a^	0.85 ± 0.04 ^a^

Parameters in columns denoted with the same letters do not differ significantly at the confidence level α = 0.05. H_0_—hardness of fresh gels (N); H_1_—hardness of gels after 24 h of storage (N); H_7_—hardness of gels after 7 days of storage (N).

## Data Availability

The data that support the findings of this study are available from the corresponding author upon reasonable request.
